# Integrated Analysis of Multiscale Large-Scale Biological Data for Investigating Human Disease 2016

**DOI:** 10.1155/2016/6585069

**Published:** 2016-09-15

**Authors:** Tao Huang, Lei Chen, Jiangning Song, Mingyue Zheng, Jialiang Yang, Zhenguo Zhang

**Affiliations:** ^1^Chinese Academy of Sciences, Shanghai, China; ^2^Shanghai Maritime University, Pudong, Shanghai, China; ^3^Monash University, Clayton, VIC 3800, Australia; ^4^Shanghai Institute of Materia Medica, Zuchongzhi Rd, Pudong, Shanghai, China; ^5^Icahn School of Medicine at Mount Sinai, New York, NY 10029, USA; ^6^Department of Biology, University of Rochester, Rochester, NY 14627, USA

With the development of high-throughput omics technologies, more and more omics data are generated. It has become common to have multiomics data for the same samples which make the integrative analysis possible. But the data integration is still challenging since there are only a limited number of methods to do such analysis. To stimulate the methodology development and applications of multiomics analysis, we collected 14 novel studies of large scale multiomics data for biomedical researches.

P. Y. De Silva and G. U. Ganegoda critically analyzed various methods used for encoding and encrypting data onto DNA and identified the advantages and capability of every scheme to overcome the drawbacks of previous methods.

J. Wu et al. integrated the MGI, GEO, and miRNA database to analyze the genetic regulatory networks under morphology difference of integument of humans and mice. And they found that the gene expression network in the skin was highly divergent between human and mouse.

L.-W. Liu L. et al. analyzed 303 samples of ovarian serous cystadenocarcinoma and the corresponding RNA-seq data. They established a risk assessment model of five genes and the AUROC value was 0.67 when predicting the survival time in testing set.

S. Wang et al. proposed a new hybrid algorithm called HICATS that incorporated imperialist competition algorithm (ICA) which performs global search and tabu search (TS) which conducts fine-tune search. The performance of their method was superior to other similar works.

J. Li et al. reviewed the paradigm of differential regulatory analysis (DRA) based on gene coexpression network (GCN). They found that DRA can reveal underlying molecular mechanism in large-scale carcinogenesis studies.

B. Liang et al. constructed a non-small cell lung cancer- (NSCLC-) specific functional association network and applied a network partition algorithm to divide the network into gene modules. From these modules, they identified NSCLC biomarkers.

B. Liu et al. developed an R package, detection of autosomal abnormalities for fetus (DASAF), which implements the three most popular trisomy detection methods—the standard *z*-score method (STDZ); the GC correction *z*-score (GCCZ) method; and the internal reference *z*-score (IRZ) method—together with one subchromosome abnormality identification method (SCAZ).

Q. Zhang et al. investigated the associations between PM2.5 and 22 disease classes, such as respiratory diseases, cardiovascular diseases, and gastrointestinal diseases. They found that several diseases, such as diseases related to ear, nose, and throat and gastrointestinal, nutritional, renal, and cardiovascular diseases, are influenced by PM2.5.

F. Wang et al. used 931 sRNA-seq datasets from the NCBI SRA database to detect and identify viruses in human cells or tissues. Six viruses including HPV-18, HBV, HCV, HIV-1, SMRV, and EBV were detected from 36 datasets and SMRV was found in Diffuse Large B Cell Lymphoma cells for the first time.

S. Wang et al. attempted to extract important features for aptamer-compound interactions using feature selection methods, such as maximum relevance minimum redundancy, and incremental feature selection. They found that quantum-chemical and electrostatic descriptors were important for aptamer-compound interaction prediction.

X.-C. Li et al. constructed oncogenetic tree to imitate the occurrence of genetic and cytogenetic alterations in human breast cancer. They found that ErbB2 copy number variation is the frequent early event of human breast cancer.

Y. Wang et al. proposed a Phylogenetic Tree-Based Motif Finding Algorithm (PMF) to analyze 16S rRNA text data. By integrating phylogenic rules and other statistical indexes for classification, it can effectively reduce the dimension of the large feature spaces generated by the text datasets.

L. Zheng et al. analyzed the subcellular localization and biological functions of Fibrillarin2, a nucleolar protein in* Nicotiana benthamiana*. They found that the protein was localized in the nucleolus and cajal body of leaf epidermal cells of* N. benthamiana* and involved growth retardation, organ deformation, chlorosis, and necrosis.

F. Yuan et al. tried to predict candidate genes related to pancreatic cancer using protein-protein interactions and a shortest path approach. The genes on the shortest path among known pancreatic cancer genes were considered as candidates that were further filtered by permutation test. Several predicted genes are promising and worth experimental validation.

With this special issue, we hope more and more people will become familiar with multiomics big data analysis and be interested in applying the integrative analysis approaches to reveal the underling mechanisms of complex biomedical phenotypes.

*Tao Huang* *Lei Chen* *Jiangning Song* *Mingyue Zheng* *Jialiang Yang* *Zhenguo Zhang*

